# Redox signaling and metabolism in Alzheimer's disease

**DOI:** 10.3389/fnagi.2022.1003721

**Published:** 2022-11-03

**Authors:** M. I. Holubiec, M. Gellert, E. M. Hanschmann

**Affiliations:** ^1^IBioBA-MPSP Instituto de Investigación en Biomedicina de Buenos Aires, Partner Institute of the Max Planck Society, Buenos Aires, Argentina; ^2^Institute for Medical Biochemistry and Molecular Biology, University Medicine Greifwald, University Greifswald, Greifswald, Germany; ^3^Independent Researcher Essen, Germany

**Keywords:** redox signaling, redox metabolism, Alzheimer's disease, neurodegeneration, Tau, APP

## Abstract

Reduction and oxidation reactions are essential for biochemical processes. They are part of metabolic pathways and signal transduction. Reactive oxygen species (ROS) as second messengers and oxidative modifications of cysteinyl (Cys) residues are key to transduce and translate intracellular and intercellular signals. Dysregulation of cellular redox signaling is known as oxidative distress, which has been linked to various pathologies, including neurodegeneration. Alzheimer's disease (AD) is a neurodegenerative pathology linked to both, abnormal amyloid precursor protein (APP) processing, generating Aβ peptide, and Tau hyperphosphorylation and aggregation. Signs of oxidative distress in AD include: increase of ROS (H_2_O_2_, O_2_^•−^), decrease of the levels or activities of antioxidant enzymes, abnormal oxidation of macromolecules related to elevated Aβ production, and changes in mitochondrial homeostasis linked to Tau phosphorylation. Interestingly, Cys residues present in APP form disulfide bonds that are important for intermolecular interactions and might be involved in the aggregation of Aβ. Moreover, two Cys residues in some Tau isoforms have been shown to be essential for Tau stabilization and its interaction with microtubules. Future research will show the complexities of Tau, its interactome, and the role that Cys residues play in the progression of AD. The specific modification of cysteinyl residues in redox signaling is also tightly connected to the regulation of various metabolic pathways. Many of these pathways have been found to be altered in AD, even at very early stages. In order to analyze the complex changes and underlying mechanisms, several AD models have been developed, including animal models, 2D and 3D cell culture, and *ex-vivo* studies of patient samples. The use of these models along with innovative, new redox analysis techniques are key to further understand the importance of the redox component in Alzheimer's disease and the identification of new therapeutic targets in the future.

## Redox signaling and metabolism

The transport of electrons within reduction and oxidation (redox) reactions is important for biochemical processes within cells. Cells possess electron carriers in the form of co-enzymes and proteins that fulfill different functions. Co-enzymes include the electron acceptors flavin adenine dinucleotide (FAD/FADH), flavin mononucleotide (FMN/FMNH), as well as nicotinamide adenine dinucleotide (NAD^+^/NADH) and the electron donor nicotinamide adenine dinucleotide phosphate (NADP^+^/NADPH). Proteins include cytochromes (containing heme), flavoproteins (containing FAD or FMN), iron-sulfur proteins (containing iron) and proteins of the Thioredoxin (Trx) family (containing Cys or Sec residue(s) within their active site motifs). Redox reactions are often part of metabolic pathways and play a role in catabolic and anabolic reactions. Therefore, they are needed for the break-down of molecules and the release of energy, as well as for the biosynthesis of complex molecules including amino acids/proteins, fatty acids/lipids and nucleotides/nucleic acids [reviewed in Hosios and Vander Heiden (2018)].

These biomolecules are known to be prone to modification and even inactivation by oxidation. In fact, first introduced in 1985, the term oxidative stress (today known as oxidative distress) has long linked non-physiological levels of reactive oxygen species (ROS) to oxidation of biomolecules and cellular structures, gene mutations, protein inactivation and aggregation and eventually clinical pathologies (Hanschmann, 2013; Sies, 2015). These reactive species can be produced exogenously or endogenously. Endogenous sources include the production by enzymes such as NADPH oxidase [NOX; generates superoxide (O_2_^•−^)], superoxide dismutases [SOD; generates hydrogen peroxide (H_2_O_2_) or NO-synthase (NOS; generates nitric oxide (NO))], and generation as by-products by enzymes or aerobic metabolism (e.g. in the respiratory chain) (Hanschmann, 2013). Organelles such as mitochondria contain specific redox networks that are linked to their specific physiological relevance (Riemer et al., 2015). Mitochondria are a major source of O_2_^•−^ and H_2_O_2_. Interestingly, mitochondrial subcompartments have distinct functions and are equipped with different sets of enzymes and redox networks. Today we appreciate the absence of a redox equilibrium and the relevance of reactive species for signal transduction and cell physiology, i.e., oxidative eustress (Hanschmann, 2013). Numerous specialized transporters and shuttles link redox metabolism to other compartments. In fact, a substrate or protein is transported in a specific redox state to another compartment, where it undergoes oxidative modification (Hosios and Vander Heiden, 2018). Many key enzymes of metabolic pathways can be regulated *via* specific and reversible oxidative modifications that act as thiol switches (Brandes et al., 2009; López-Grueso et al., 2019; Gao et al., 2020). They are part of redox signaling circuits and depend on i) second messengers like H_2_O_2_, hydrogen sulfide (H_2_S), and NO, and ii) the catalysis by enzymes of the Trx family (including thioredoxins, glutaredoxins, and peroxiredoxins) (Hanschmann, 2013).

## Redox in Alzheimer's disease

Alzheimer's disease (AD) is a neurodegenerative pathology that has both neurological and cognitive effects (Knopman et al., 2021). AD is first evidenced in two main brain areas: the entorhinal cortex (EC) and the hippocampus (Leng et al., 2021). Like other neurodegenerative diseases, AD presents with selective vulnerability (Leng et al., 2021). Recent studies state that early dysregulation of signaling pathways in the EC include alterations in the redox signaling and neuroinflammation (Olajide et al., 2021). Redox changes and alterations of mitochondrial homeostasis seem to be of utmost importance for the early development of the AD pathology in the EC. Different neuronal populations present distinct sensitivities toward oxidative damage (Wang and Michaelis, 2010), e.g., damage to macromolecules in the early stages of AD was detected in EC neurons, which are particularly sensitive to oxidative damage (Terni et al., 2010). Furthermore, distinct neuronal populations in the hippocampus respond differently to increased oxidative conditions, particularly the CA1 neurons are greatly affected (Wang et al., 2005; Olajide et al., 2021). Redox changes, such as an increase in H_2_O_2_, RNA oxidation observed in samples from patients, and mitochondrial dysfunction, that was evidenced as an increase of lipid peroxidation of the α subunit of the mitochondrial ATP-synthase, seem to be of utmost importance for the early development of the AD pathology in the EC (Nunomura et al., 2001; Terni et al., 2010; Olajide et al., 2021).

**Figure 1:**
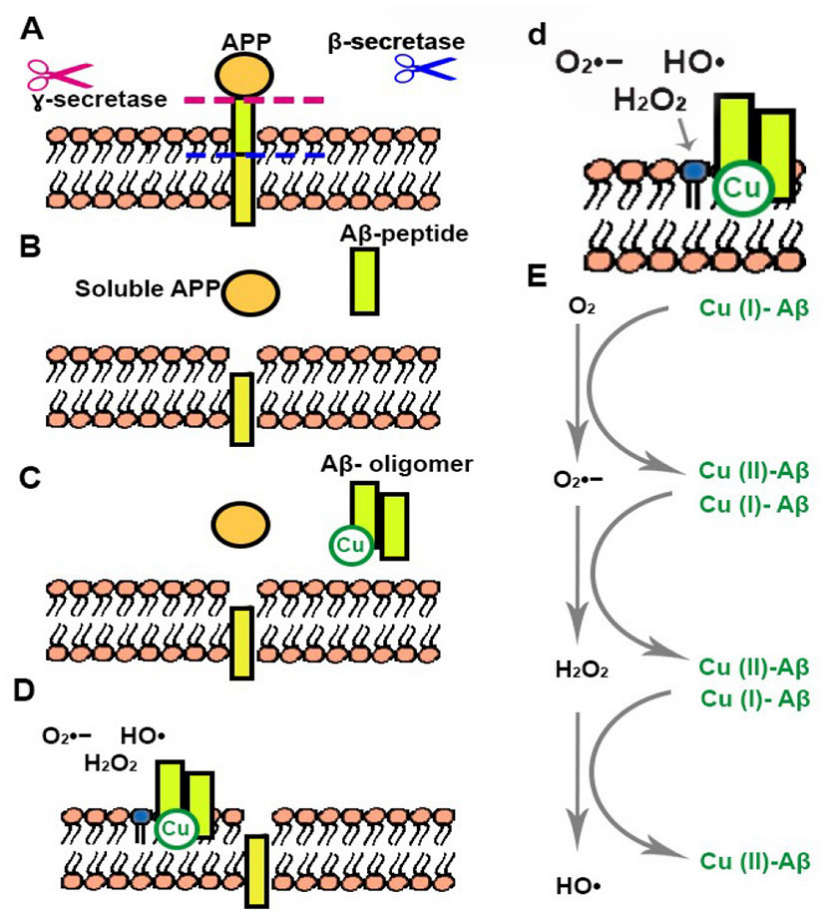
Generation of Aβ peptides and Cu related ROS production. **(A)** Aβ peptide is produced from the proteolysis of APP that takes place through the action of β- and γ-secretases. **(B)** Aβ peptide and soluble APP are released. **(C)** Aβ peptide forms oligomers that can interact with Cu. **(D,d)** Aβ oligomer-Cu complexes are incorporated into the cellular membrane generating changes in nearby phospholipids. **(E)** Aβ oligomer-Cu complexes can be oxidized in the presence of O_2_, generating different ROS.

The most known hallmarks of AD in patients are neurofibrillary tangles (NFT) and amyloid beta (Aβ) deposits. NFTs are mainly conformed by phosphorylated Tau proteins that aggregate in intracellular tangles. Aβ deposits are extracellular aggregates generated from the Aβ-peptide (Figure 1A). These are produced from the proteolysis of the amyloid precursor protein (APP) that takes place through the action of two different secretases (β- and γ-), generating different peptides among which Aβ-40 and Aβ-42 are of utmost relevance in AD (Figures 1A,B). An increase of positive β-secretase levels has been observed in the presence of high levels of peroxides (Tamagno et al., 2008). APP cleavage by α-secretase initiates the non-amyloidogenic pathway, the primary α-secretase is a metalloprotease called ADAM10 that is also involved in the regulation of redox related proteins, such as Trx1 cleavage and generation of the secreted Trx80 (Kuhn et al., 2010; Gil-Bea et al., 2012; Swomley et al., 2014), as well as being subject to activation through disulfide isomerization depending on oxidative conditions (Atapattu et al., 2016). Tau is a microtubule-associated protein (MAP) that, along with other proteins, dynamically interacts with neuronal microtubules, stabilizing them under physiological conditions (Maccioni and Cambiazo, 1995; Avila et al., 2004; Pîrşcoveanu et al., 2017), allowing dynamic changes in the extremes of these elements (Peña-Ortega et al., 2022). Under certain circumstances Tau undergoes posttranslational modifications that may result in the generation of oligomers and aggregates in neurons (Avila et al., 2004). The toxicity of Aβ and Tau aggregates has been thoroughly studied during the last years and was linked to changes in metabolism and the production of ROS, such as ${\text{O}}_{2}^{•-}$, H_2_O_2_ and ^•^OH (Carrillo-Mora et al., 2014; Yan and Wang, 2014; Cheignon et al., 2017). However, these metabolic and redox changes could be related to other consequences of AD. For instance, it has been proven that microglial activation leads to inflammation and an increase of ROS such as ${\text{O}}_{2}^{•-}$ produced by NOX (Simpson and Oliver, 2020). NOX is a group of seven enzymes that particularly produce ${\text{O}}_{2}^{•-}$ upon activation. Two isoforms (NOX2 and 4) have been identified in microglial cells and they are activated upon neuroinflammation or neurodegeneration (Simpson and Oliver, 2020). ROS, such as H_2_O_2_ or ${\text{O}}_{2}^{•-}$, are important second messengers and as such modulators of the immune response by activating signaling pathways in microglia, e.g., H_2_O_2_ activates NFκB signaling (Kim et al., 2008).

Other AD related cellular changes include astrogliosis, dystrophic neurites and synapse and neuronal loss (Selkoe, 2001). The relation between neuronal survival and redox regulation has been thoroughly studied and reviewed in recent years (Satoh and Lipton, 2007; Sabens Liedhegner et al., 2012; Sultana et al., 2013; Yin et al., 2014; Sbodio et al., 2019). For instance, an increase of ROS gives rise to cysteine modifications. Reversible protein mixed disulfides with glutathione (GSH) may be the most common steady-state derivative, since GSH is abundant in the cell. Thus, S-glutathionylation may act in redox signal transduction and glutaredoxins (Grxs) serve not only as reducing enzymes upon neuroinflammation but are also involved in the catalysis of (de-)glutathionylation (Sabens Liedhegner et al., 2012; Hanschmann, 2013). Grx1 and Grx2 are expressed in the brain (Aon-Bertolino et al., 2010; Godoy et al., 2011). It has been shown that neurons that present different levels of Grx also show a different viability response in AD. Immunohistochemical analysis showed lower Grx1 and 2 levels in neurons presenting signs of neurodegeneration compared to healthy neurons (Akterin et al., 2006; Arodin et al., 2014). Furthermore, higher levels of Grx1 and Trx1 have been found in plasma and cerebrospinal fluid of AD patients (Arodin et al., 2014). Interestingly, there are no apparent changes in Trx1 levels in the brain. However, there is a decrease in Trx2 immunostaining in the axons of the CA1 hippocampal area in AD patients.

Uric acid, bilirubin, lycopene, α and β carotene, vitamin A, vitamin C, and vitamin E, present decreased levels in patients with AD (Foy et al., 1999; Kim et al., 2006). Even though initial works have shown that some antioxidant enzymes present a greater activity in cells from AD patients (Zemlan et al., 1989), it has recently been shown that many antioxidant enzymes, such as SOD2, catalase and glutathione peroxidase, present lower activities in AD (Marcus et al., 1998; Omar et al., 1999; Wang et al., 2014). A study performed in patients has shown that low GSH levels in plasma lead to a lower risk of AD development (Charisis et al., 2021). Furthermore, a study performed in mice showed that the increase of SOD levels could diminish some of the characteristic symptoms of AD including learning and memory deficits (Massaad et al., 2009).

All in all, the pathophysiology of AD goes beyond Aβ aggregates and Tau neurofibrils and it is linked to a complex network of redox regulation and signaling that should be the focus of further investigation.

## Sporadic and familial Alzheimer's disease

There are two major forms of AD that present particular characteristics albeit the same symptoms (Piaceri et al., 2013). Sporadic AD has a late onset and the etiology of the disease is not clear. Environmental and genetic causes have been pinpointed as key to this form of AD. It is generally known that AD is tightly related to advanced age, this being one of the main risk factors in sporadic AD (Katzman, 1988; Riedel et al., 2016; Armstrong, 2019). The relation between oxidative damage and aging has been previously addressed (Muller et al., 2007; Sanz and Stefanatos, 2008). This relation has received some criticism since there are many examples that contradict the fact that a higher production of free radicals is directly related to longevity. However, subsequent and complex studies have pointed to senescence pathways as key players in the relation between aging and redox regulation (Chandrasekaran et al., 2017). Mitochondrial metabolism, bioenergetics and redox signaling are of utmost importance in neurodegeneration and the progression of AD (Yap et al., 2009; Yin et al., 2014). Some areas of the brain, such as the hippocampus, are more susceptible to lower cell excitability, changes in synaptic plasticity and diminished synaptic transmission (Kumar et al., 2018). It was shown that increased oxidative conditions in neurons can be an early marker of Alzheimer's disease and, through molecular signaling, it may contribute to impaired synaptic function (Foster et al., 2017).

Familial AD (FA) occurs in many patients at an earlier onset and has a clear etiology. It is caused by mutations in three specific genes: APP (Amyloid Beta Precursor Protein), PSEN1 (Presenlin1) and PSEN2 (Presenilin2) (Piaceri et al., 2013). FA was firstly associated with mutations in APP and has been shown to increase Aβ-42 levels compared to Aβ-40 (Finckh et al., 2005; Munter et al., 2010). Autosomal FA may account for ~0.5% of AD cases (Finckh et al., 2005). Although most of the mutations causing FA are known to generate an increase in Aβ-42 levels, some mutations that occur within the APP GxxxG motif can cause a decrease in this form of the peptide (Munter et al., 2010). Mutations in APP, PSEN1 and PSEN2 are responsible for the increase in Aβ-peptide production in patients and cause the same symptoms and cellular changes as described for sporadic AD (Finckh et al., 2005). Thus, the study of the impact of these specific mutations can shed light on the etiology of AD (Selkoe, 2001).

## Aβ and APP, and their role in redox regulation in AD

Mutations in APP, PSEN1 or PSEN2 have been shown to lead to increased production of Aβ peptide (Gaitonde et al., 1975; Sultana et al., 2009), and these are closely linked to FA. Regarding presenilin, changes in different Cys residues present in PSEN1 have been related to the onset of FA. Of these two, Cys92 located at the N-terminus is highly conserved and it has been shown that its mutation leads to an increase of Aβ-42 in HEK293 cells (Zhang et al., 2000; Tandon and Fraser, 2002). Mutations in PSEN2 are not so common in FA or AD patients and the described mutations do not include changes in Cys residues (Tandon and Fraser, 2002). Nevertheless, mutations near a Cys residue can also affect protein regulation and function, and these should be taken into further consideration. It is worth noting that APP presents a Cu binding site and that it has been shown that incubation of APP with Cu (II) results in the reduction to Cu (I) and the oxidation of Cys144 in APP (Multhaup et al., 1996; Ruiz et al., 1999; Barnham et al., 2003a; Kong et al., 2008). Binding Cu leads to oxidative modification of APP. Moreover, the reduction of Cu can give rise to the formation of ROS, such as ${\text{O}}_{2}^{•-}$ or hydroxyl radicals (^•^OH) (Ruiz et al., 1999). The production of ROS by Cu-APP can be linked to theories that state that AD arises well before the formation of Aβ and that modifications in APP are the main cause (Multhaup et al., 1996). In APP full length, Cys144 forms a disulfide bond with an additional Cys residue in the protein, that could belong to a Cys-rich domain present at the N-terminus of APP, but was not clearly identified (Multhaup et al., 1996; Zhang et al., 2021). Peptides with only Cys144 form intermolecular disulfide bonds creating dimers (Multhaup et al., 1996). Zhang et al. described a rare mutation in APP, near the Cys-rich domain, that generates an increase in Aβ production related to a fast exit of APP from the endoplasmatic reticulum (ER) (Zhang et al., 2021). Interestingly, the palmitoylation of Cys 186 and 187 was shown to be of importance for APP release from the ER, leading to more amyloidogenic processing and Aβ production (Bhattacharyya et al., 2013). Furthermore, this Cys cluster appears to be important for APP dimerization, which decreases APP localization to the plasma membrane, thus diminishing amyloidogenic processing (Ciuculescu et al., 2005; Baumkötter et al., 2014). These studies point to a clear relation between APP (and its processing) and redox regulation, ranging from the presence of highly conserved Cys residues in APP and PSEN1 to the increased generation of different ROS (${\text{O}}_{2}^{•-}$, ^•^OH, H_2_O_2_).

There are many studies linking Aβ with oxidation and toxicity, explaining how mutations that produce larger amounts of this peptide can lead to an early onset development of AD (Barnham et al., 2003b; Everett et al., 2014; Cheignon et al., 2017; Elsworthy et al., 2021). Increased oxidation of macromolecules, such as nucleic acids, proteins and lipids, has been related to higher levels of Aβ-40 and Aβ1-42 in AD hippocampus and cortex (Butterfield and Lauderback, 2002). It has been shown that Aβ peptide can reduce metal ions such as iron (Fe) and copper (Cu) generating ^•^OH radicals through the Fenton reaction (Sbodio et al., 2019). Aβ aggregation leads to the generation of β-sheet rich structures composed of oligomeric species that are reorganized into protofibrils and fibrils (Figure 1C) (Finder and Glockshuber, 2007; Pham et al., 2010; Forloni et al., 2016; Cheignon et al., 2017). Aβ oligomers can impair Cys uptake and GSH synthesis through excitatory amino acid transporter 3 (EAAT3) inhibition (Hodgson et al., 2013). This transporter plays a critical role in neuronal redox regulation, and its depletion can promote oxidative distress and neurodegeneration (Hodgson et al., 2013). However, the most accepted hypothesis to this day states that Aβ oligomers that accumulate in the central nervous system are of utmost toxicity for cells since they can interact with lipids and permeabilize cellular membranes (Figures 1D,d), leading to cell dysfunction, and neurodegeneration (Cheignon et al., 2017). The permeabilization of membranes by Aβ-42 oligomers has been reported as a common component of amyloid toxicity making the disruption of neuronal membrane biomolecules a common feature of AD (Figures 1D,d) (Schubert et al., 1995; Glabe, 2006). The hydrophobicity of some of the aggregates allows them to incorporate into the lipid bilayer initiating the production of ROS, mainly O_2_^•−^, ^•^OH, and H_2_O_2_ leading to the oxidation of lipids and membrane proteins (Figures 1D,E) (Fraser et al., 1993; Stege and Bosman, 1999; Schmidt et al., 2009; Butterfield et al., 2013; Evangelisti et al., 2013; Swomley et al., 2014; Yan and Wang, 2014). Cu is capable of cycling between two redox states, which makes it a redox-active ion (Drommi et al., 2021). It has been suggested that the high levels of Cu present in senile plaques are related to covalent crosslinking of Aβ. Cu interaction with Aβ in these structures could be responsible for the formation and stabilization of oligomers and aggregates (Figures 1D,d) (Atwood et al., 2004; Chassaing et al., 2012; Drommi et al., 2021). Since Cu can be incompletely oxidized it can lead to the formation of ROS such as O_2_^•−^, ^•^OH, H_2_O_2_, increasing oxidation and damage to macromolecules (Figure 1E) (Drommi et al., 2021). Recent studies suggest a mechanism mediated by methionine-35 (Met35) of Aβ-42 in the process of ROS production and oxidation (Swomley et al., 2014; Friedemann et al., 2015). Briefly, Aβ-42 oligomers re-enter the bilayer acquiring an alpha-helical structure in which Met35 would interact with the carbonyl of ilein-31 allowing the priming of the electrons in the methionine (Friedemann et al., 2015). In this hydrophobic environment the primed Met stabilizes as sulfuranyl free radical that is able to oxidize thiols or ascorbate and increases ${\text{O}}_{2}^{•-}$ and H_2_O_2_ levels (Schöneich, 2002; Friedemann et al., 2015). In addition to the oxidation of Met35, tyrosine can also be oxidized in Aβ peptides, to form a dityrosine covalent dimer (Al-Hilaly et al., 2013). Different studies have shown that aggregates present this dityrosine dimer, linking this oxidation to the formation of neurofibrils (Atwood et al., 2004; Al-Hilaly et al., 2013). Furthermore, it has been shown that lack of methionine sulfoxide reductase A-1 (MsrA-1), that specifically reduces oxidized methionines in protein targets, produces a decrease in amyloid aggregates in a Caenorhabditis elegans model, and tips the balance toward an increase of oligomeric aggregates, correlating with an increased synaptic dysfunction (Minniti et al., 2015). On this note, the mainstream ideas on Aβ aggregation suggest that amyloid aggregates present less toxicity than oligomeric ones (Uddin et al., 2020; Koike et al., 2021). However, the impact of Aβ oxidation on its aggregation is still controversial. Some studies state that oxidation of Met35 impedes amyloid aggregate formation (Hou et al., 2013; Pilkington et al., 2019), while others propose that it increases aggregation of the peptide (Maiti et al., 2021).

All in all, Aβ plays a key role in the generation of increased levels of oxidation. However, the pathways through which this occurs are still not clear and further studies focused on the molecular production of specific ROS seems to be essential.

## Mitochondrial dysfunction in AD

**Figure 2:**
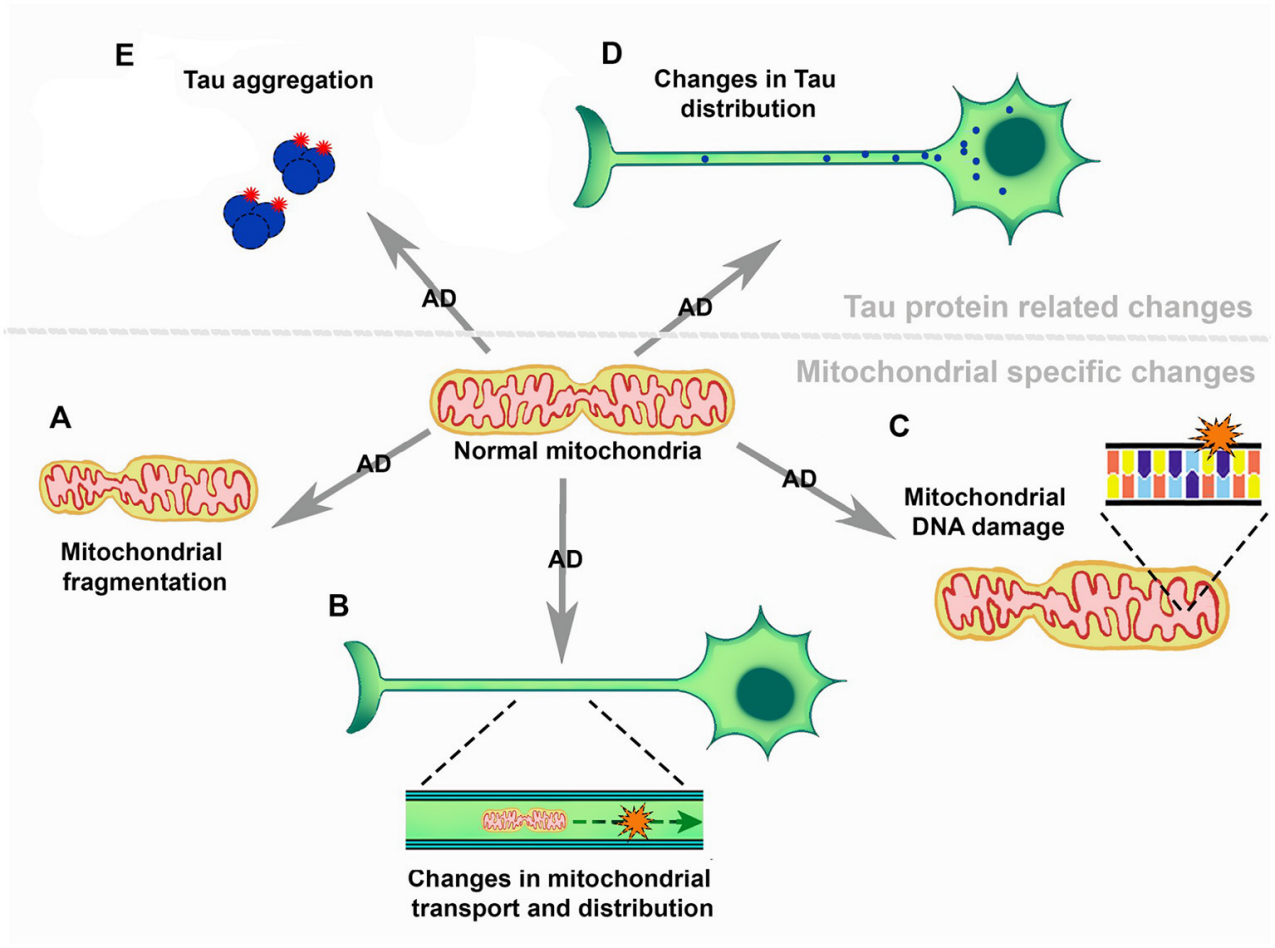
The role of mitochondria in the development and progression of Alzheimer's disease. **(A)** Mitochondrial fragmentation. **(B)** Mitochondrial transport and distribution across neurons. **(C)** Mitochondrial DNA damage. **(D)** Changes in Tau distribution and **(E)** Tau aggregation.

Mitochondria function in ATP generation, a process known as oxidative phosphorylation (Dikalov, 2011). During this process electrons, supplied by NADP or succinate, are transferred through different complexes of the respiratory chain (Dikalov, 2011). Here, ${\text{O}}_{2}^{•-}$ production occurs at the ubiquinol oxidation center of complex III and the FAD site of complex II. Also, both ${\text{O}}_{2}^{•-}$ and H_2_O_2_ are produced at the 2-oxoacid dehydrogenase complexes (Wang et al., 2014; Larosa and Remacle, 2018). Previous reviews delve into mitochondrial altered dynamics and morphology in AD (Wang et al., 2009a; Misrani et al., 2021). Mitochondrial dysfunction has been linked to Aβ and Tau (Meng et al., 1990; Calkins and Reddy, 2011; Manczak and Reddy, 2012; Manczak et al., 2016). Interestingly, alterations and functional changes in mitochondria appear before the formation of Aβ plaques or Tau neurofibrils (Calkins et al., 2011; Correia et al., 2016). Briefly, changes in mitochondrial fission and fusion occur in AD, and some findings suggest that this dynamical balance is tipped toward less fission in AD (Wang et al., 2008a). On the contrary, several analyses suggest that there is an increase of mitochondrial fragmentation in AD (Figure 2A), that have been ascribed to Aβ toxicity (Wang et al., 2008b, 2009b, 2017). These alterations in the fusion/fission balance are clearly related to mitochondrial morphological changes that have been observed in AD (Collins, 1990; Wang et al., 2008a, 2009b; Flannery and Trushina, 2019). Importantly, during the early stages of AD there is evidence of changes in mitochondrial axonal transport (Figure 2B) (Correia et al., 2016; Flannery and Trushina, 2019). Changes in mitochondrial transport could be the cause of early axonal degeneration and the beginning of neuronal loss (Calkins et al., 2011; Guo et al., 2013; Correia et al., 2016). There is a decrease in mitochondrial DNA coupled with mitochondrial DNA damage, mediated by 8-oxo-7,8-dihydro-29-deoxyguanosine (8-OhdG) accumulation in patients with AD (Figure 2C) (de la Monte et al., 2000; Brown et al., 2001). This points to lower expression of mitochondrial proteins, particularly some components of the respiratory chain, such as cytochrome oxidase (COX) in complex IV (Brown et al., 2001). The activity of several mitochondrial enzymes, such as the pyruvate dehydrogenase complex, the α-ketoglutarate dehydrogenase complex and the respiratory complex IV, is reduced in AD (Parker et al., 1994; Fišar et al., 2019). However, different works have reported somewhat contrary results regarding mitochondrial protein expression (Silva et al., 2012). Some studies have shown an increase in COX levels (Hirai et al., 2001) while others have evidenced a decrease of this enzyme (Nagy et al., 1999; de la Monte et al., 2000). Manczak et al. suggested that an increase in these protein levels could be a compensatory mechanism since they found that in the same samples that showed increased expressions of COX 1 and 2 there was decreased downregulation of the subunit 3 in complex III and of ATPase 6 and 8 in complex V (Manczak et al., 2004). A transcriptomic study using neurons from donor patients suffering from AD, coupled with western blot analysis, showed that there is a lower expression of genes related to the mitochondrial electron transport chain. This study suggests a relation between lower cerebral metabolic rates and the reduction in the expression of genes encoding for the electron transport chain (Liang et al., 2008).

There are several mitochondrial alterations in AD, ranging from fragmentation to mitochondrial DNA damage (Figure 2). It is known that the mitochondria play a key role in redox metabolism and regulation, and present an association to neurodegenerative diseases such as AD. However, the studies focused on this topic are scarce and they are not particularly focused on mitochondrial homeostasis and redox metabolism in AD.

## Tau toxicity, and its role in oxidation and redox regulation

Tau is a complex protein with intrinsically disordered regions, that presents 6 different isoforms in the CNS, two of these depend on the inclusion and exclusion of exon 10, generating 3R Tau and 4R Tau isoforms (Bachmann et al., 2021). In healthy human brains 3R/4R Tau levels are present at a 50/50 ratio (Chen et al., 2010). Mutations in the Microtubule Associated Protein Tau (MAPT) gene, which encodes the Tau protein, are known to be related to a number of neurodegenerative diseases (Guo et al., 2017; Cherry et al., 2021; Esteras et al., 2022). Mutations altering exon 10 splicing can increase the levels of 4R or 3R Tau which are also related to several neurodegenerative diseases, e.g., fronto temporal dementia and progressive supranuclear palsy (Chen et al., 2010; Qian and Liu, 2014; Bachmann et al., 2021; Stamelou et al., 2021). NMDA activation can generate ROS, particularly O_2_^•−^ (Brennan et al., 2009; Girouard et al., 2009; Esteras et al., 2022). Inversely, NMDA receptors can be modulated by the cellular redox state, altering synaptic functions in AD (Bodhinathan et al., 2010; Guidi et al., 2015). Esteras et al. (2022) demonstrated that 4R Tau generates changes in NMDA receptor activity and that these changes can be modulated by mitochondrial antioxidants, such as MitoQ. This drug mimics the endogenous mitochondrial antioxidant coenzyme Q10 activity and augments the enzyme capacity (Tauskela, 2007). Similarly, hyperphosphorylated Tau has been shown to interact with the mitochondrial Dynamin-related protein 1 (Drp1), affecting mitochondrial fission and fusion. Decreased levels of Drp1 protect against mitochondrial alterations generated by Tau (Manczak and Reddy, 2012; Kandimalla et al., 2016). Cofilin-1 is a redox regulated protein that plays a key role in mitochondrial function (Kang and Woo, 2019). Upon oxidation, intermolecular disulfide bonds form and Cofilin loses its affinity for actin. Cofilin then translocates to the mitochondria, where it generates a drop in mitochondrial membrane potential, and cytochrome c release through promotion of the opening of the permeability transition pore (Kang and Woo, 2019). A very recent and interesting work has shown that a cluster of mitochondria belonging to the axon initial segment, and with particular characteristics and little motility, is important for Tau sorting (Figure 2D) (Tjiang and Zempel, 2022). It has been shown that ROS produced in mitochondria, such as H_2_O_2_, can generate Tau oligomer formation in a mouse model (Figure 2E) (Du et al., 2022). Moreover, inhibition of GSH synthesis in a cell model produced an increase in tau phosphorylation, a first step toward Tau oligomer formation (Su et al., 2010). Also, Tau 4R presents two Cys residues that seem to be important for Tau pathology development in a Drosophila Taupathy model (Prifti et al., 2021). These Cys residues seem to be important for Tau stability and its interaction with microtubules, and Cys-322 can affect tau aggregation (Prifti et al., 2021). Cys-322 as well as Cys-291 appear to be important for the polymerization of human Tau (Bhattacharya et al., 2001; Chen et al., 2018). Other works in Dropsophila melanogaster have shown that the substitution of these Cys diminishes 4R Tau toxicity and they contribute to Tau accumulation under oxidative conditions (Saito et al., 2021).

There are few studies linking modifications in Tau to the onset of AD. Some works mention Lys modifications, such as succinylation (in Tau, APP, and mitochondrial proteins) (Yang et al., 2022), tau acetylation (Min et al., 2010) that, in fact, can be linked to redox regulation (Lucke-Wold et al., 2017) and APP acetylation (Bai et al., 2022). The presence of Cys residues that are important for Tau polymerization led us to believe that redox regulation and signaling of and *via* this protein are extremely important in AD, since one of the clearest hallmarks of the disease is Tau aggregation into neurofibrils. Furthermore, modifications in Tau aggregation and localization are related to mitochondrial homeostasis. This link should be further explored in the future.

It is important to mention that Tau is a complex protein that does not only bind microtubules. Recent studies have found that both tau and p-tau present multiple interaction partners, some of which are located in the mitochondria and are related to redox homeostasis (Drummond et al., 2020; Sinsky et al., 2020; Jiménez, 2022; Tracy et al., 2022). Drummond et al. analyzed the interactome of p-tau inclusions dissected from AD patient brains. It is interesting to note that among the p-tau interaction partners, mitochondrial proteins such as Cytochrome c oxidase subunit 5B and Aldehyde dehydrogenase were found (Drummond et al., 2020). Moreover, an interaction with Prx5, a member of the Thioredoxin family proteins, was found (Drummond et al., 2020) clearly linking tau and its phosphorylated form not only to mitochondria but also to redox regulation in AD. Tracy et al. (2022) used a very clever approach with multiple tau mutations that cause fronto temporal dementia, showing that these mutations affect tau interaction with several mitochondrial proteins and impair metabolism and bioenergetics. Tau has not only been shown to interact with other proteins but also with DNA, making it less susceptible to peroxidation (Wei et al., 2008). A list of redox-relevant Tau interaction partners can be found in Table 1.

**Table 1:** List of redox regulated Tau interaction partners, their function(s), localization, as well as a link to redox regulation and metabolism.

**Protein**	**Function**	**Localization**	**Redox-regulation/metabolism**	**Relevance in neurodegeneration**	**References**
AKT1	Serine/threonine-protein kinase, regulates major, cellular processes e.g., cell growth, proliferation	Cytoplasm, nucleus, cell membrane	Redox-sensitive Cys residues; oxidized by hydrogen peroxide; regulated by Grx1	Related to Parkinson's disease (mouse model)	(Murata et al., 2003; Durgadoss et al., 2012; Su et al., 2019)
APOE	Lipoprotein-mediated lipid transport/clearance	Cytoplasm	Redox status of Cys affects ApoE lipid interactions	APOE is related to Sporadic Alzheimer‘s	(Marcel et al., 1983; Arnon et al., 1991; Strittmatter et al., 1994; Krimbou et al., 2004; Yamauchi and Kawakami, 2020)
APP	Neurite growth, neuronal adhesion and axoneogenesis	Cell membrane	See chapter “Aβ and APP, and their role in redox regulation in AD”	Involved in Familiar Alzheimer's	(Satpute-Krishnan et al., 2006; Seamster et al., 2012; Baumkötter et al., 2014; Maron et al., 2020)
TOMM40	Mitochondrial transport	Mitochondria	Mitochondrial function and homeostasis	Involved in Alzheimer's disease	(Siddarth et al., 2018; Lee et al., 2021; Tracy et al., 2022)
ATP5H	ATP synthase subunit	Mitochondria	Mitochondrial metabolism	RPE neurodegeneration, Spinocerebellar ataxia type 1	(Song et al., 2018; Chaphalkar et al., 2020; Tracy et al., 2022)
PRX5	Reduction of H_2_O_2_	Mitochondria	Peroxidase and sensor/regulator of peroxides	Related to Chorea-Acanthocytosis, neuronal death, microglial activation	(Rhee et al., 2012; Park et al., 2016a,b; Lee et al., 2020; Federti et al., 2021; Tracy et al., 2022)
PRX6	Reduction of H_2_O_2_	Cytosol	Peroxidase and sensor/regulator of peroxides	Involved in Alzheimer's disease- regulation of serotonergic pathway	(Rhee et al., 2012; Park et al., 2016a; Pankiewicz et al., 2020; Gu et al., 2022; Tracy et al., 2022)
FYN	Tyrosine-protein kinase	Cytoplasm, Cell membrane	Related to Nrf2 activation.	Neuroinflammatory pathways	(Dong et al., 2022; Marotta et al., 2022)
GSK3B	Protein Kinase	Cytoplasm, Cell membrane	Related to glucose metabolism and homeostasis	Related to Alzheimer's and Parkinson's disease	(Lei et al., 2011; Sacco et al., 2019; Sun et al., 2020)
COX5B	Cytochrome oxidase subunit	Mitochondria	Respiratory chain enzyme	Involved in Alzheimer's disease (mouse model)	(Drummond et al., 2020; Huang et al., 2021)

## Redox metabolism in AD

Several metabolic alterations have been described in different AD models (Toledo et al., 2017). A general overview about metabolic pathways in AD-related pathologies, i.e., lipid, glucose, tryptophan, purin, vitamin, and metal ion metabolism, and the involvement of redox signaling in these pathways is given in (Chen et al., 2020). Glucose metabolism in the CNS has been found to be impaired in AD patients in early stages of the disease (Chen and Zhong, 2013). It has been shown that there is a correlation between AD and its severity with elevated glucose concentrations in the CNS, reduced glycolytic flux, and lower levels of GLUT1 and GLUT3 (An et al., 2018; Han et al., 2021). Besides impaired glucose uptake, a rise in inactive pyruvate dehydrogenase (PDH) and increased aerobic glycolysis and ketone body metabolism were observed in different AD models and patients (Han et al., 2021). Redox active α-lipoic acid, which is one of the co-factors of pyruvate dehydrogenase, may have neuroprotective effects. Research on the molecular effects and therapeutic potential of α-lipoic acid was recently summarized in (Kaur et al., 2021). Interestingly, under oxidative conditions, such as high H_2_O_2_ concentrations, the α-lipoic acid in α-ketoglutarate dehydrogenase, an enzyme similar to the PDH, the activity of which is also decreased in AD, is reversibly glutathionylated thereby protecting the sulfur-containing compound from modification e.g., by 4-hydroxynonenal (Applegate et al., 2008). Impaired pyruvate metabolism leads to the deficiency of acetyl CoA as an energy source, also causing a decrease in acetylcholin synthesis (Kaur et al., 2021). Although Tau acetylation has been linked to AD, there is only little evidence of a connection between this modification and metabolic changes. Shin et al. described that increased NAD levels decrease Tau acetylation and neurodegeneration (Shin et al., 2021). A recently published acetylome analysis in AD brains has shown that there are acetylation changes mainly in mitochondrial proteins related to ATP synthesis and proton transport (Sun et al., 2021). In addition, mitochondrial homeostasis is altered in AD (see “Mitochondrial dysfunction in AD”). Changes in the succinylation of Lys residues in samples from AD patients occur mainly in the mitochondria and these changes have been linked to mitochondrial metabolic dysfunction (Yang et al., 2022). However, Glucose is not only most important as energy source but is substrate to the pentose phosphate pathway (PPP). Already in 1999 it was discovered, that the key enzyme of the PPP, the glucose-6-phosphate dehydrogenase, is upregulated in AD (Russell et al., 1999). Both parts of the PPP, the oxidative and non-oxidative part, are active in neurons even though to different degrees depending on the cell type (Brekke et al., 2012). The reducing equivalents generated in the PPP are essential for the function of redox enzymes including the Trx and Grx/GSH systems.

**Figure 3:**
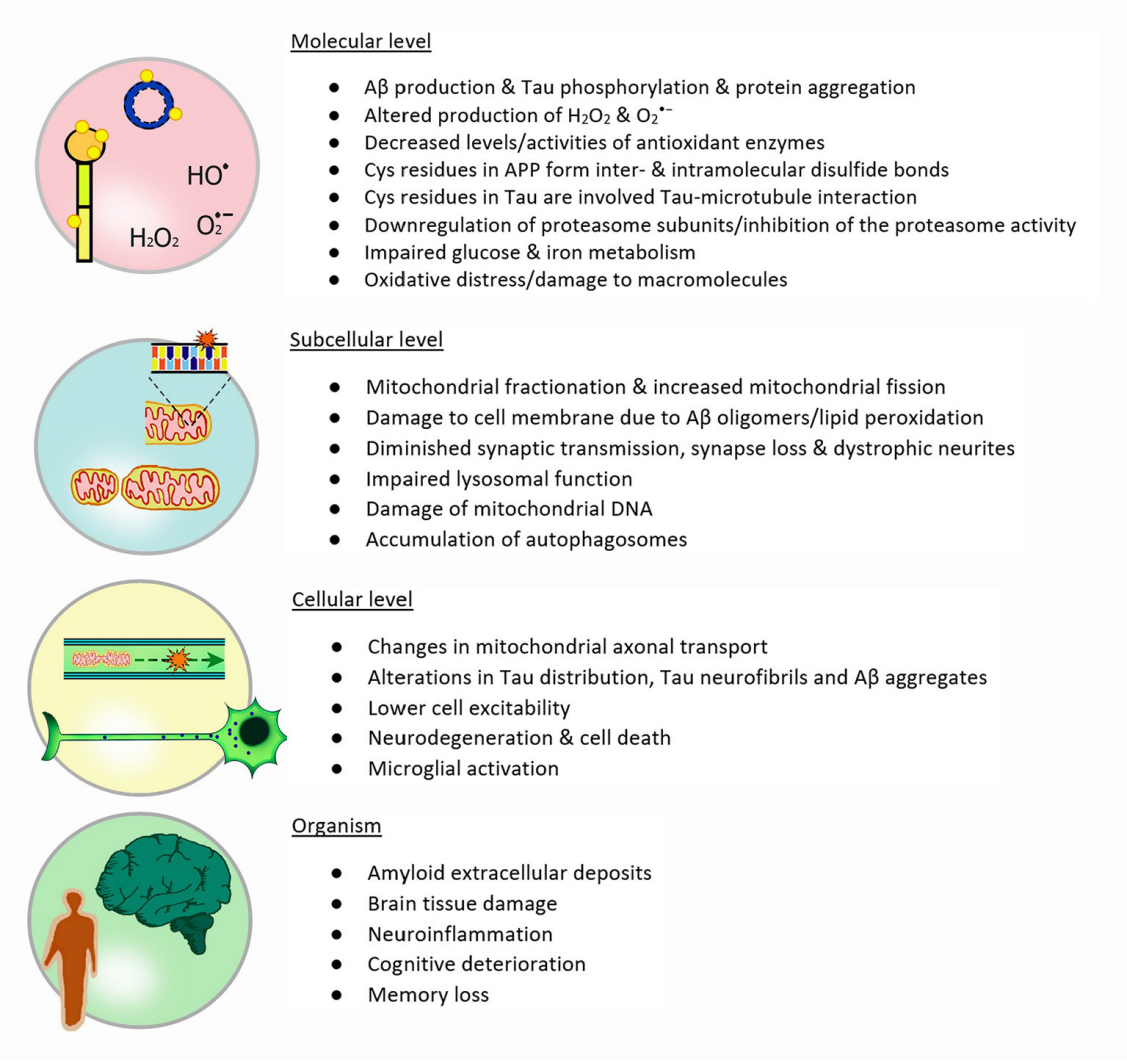
Redox metabolism in AD: The illustration depicts the four different levels of redox and metabolic changes in Alzheimer's disease: Molecules involved and their importance, different subcellular and functional changes, cellular alterations and finally the impact on the whole organism and particularly in patients. Each stage presents features that are a direct or indirect consequence of events described in the previous level.

Besides glucose metabolism and mitochondrial dysfunction, the impaired ability of a controlled protein metabolism plays an important role in AD. The downregulation of proteasome subunits as well as the inhibition of the proteasome activity, e.g., by Tau polymers, leads to the accumulation not only of damaged and missfolded proteins but also hyperphosphorylated Tau (Graham and Liu, 2017). Additionally, the lysosomal degradation of proteins and organelles is impaired through a byproduct of lipid peroxydation (4-hydroxynonenal), leading to the accumulation of autophagosomes and polyubiqinated proteins (Zhang et al., 2017). It was also suggested that this loss of protein degradation *via* the proteasome occurs already at the earliest stages of AD (Cecarini et al., 2007). Lipid peroxidation products such as hydroxynonenal and malondialdehyde may be the result of an impaired iron metabolism thereby inducing neurodegeneration (Stockwell et al., 2017). There is evidence that treatment with mesenchymal stem cell leads to a decrease in malondialdeyde and phosphorylated tau aggregates (Abozaid et al., 2022). Elevated iron levels may be one source for hydroxyl radicals as it reacts easily with H_2_O_2_ in the Fenton reaction. A number of features found in neurodegenerative diseases including AD are consistent with iron induced cell death, i.e., ferroptosis (Stockwell et al., 2017; Wang et al., 2022). The importance of iron metabolism and ferroptosis in neurodegeneration is summarized e.g., in (Stockwell et al., 2017; Li et al., 2022; Wang et al., 2022). Next to high iron levels, glutathione is significantly decreased in the hippocampus of AD patients (Mandal et al., 2022). At the same time the amount of S-glutathionylated proteins is elevated in AD brains already at early stages and especially in the hippocampus (Zhang et al., 2012). For an overview of alterations observed in AD from a molecular level to their effects on the organism see Figure 3.

## Redox tools for *in vivo* analysis of Alzheimer's disease–Technical obstacles and limitations

Suitable experimental models, including animal models, organoids, and cell culture models, are the key to gaining a better understanding of the pathogenesis of diseases such as Alzheimer's disease as well as to identify new potential therapeutic approaches (Drummond and Wisniewski, 2017; Esquerda-Canals et al., 2017; Papaspyropoulos et al., 2020). Transgenic mice constitute the animal model in AD research that is used the most. Overviews of the most common transgenic mouse and rat models of AD with indicated mutations are summarized in (Drummond and Wisniewski, 2017; Esquerda-Canals et al., 2017). The use of non-human primates has the benefit that they share a 100% sequence homology with human Aβ. Rhesus monkeys are the most practical primate model with an age-related Aβ pathology (Drummond and Wisniewski, 2017). Other species with age-related AD associated pathologies are dogs and the Octodon degu but their use has practical and scientific limitations, i.e., long lifespans and variable pathologies (Drummond and Wisniewski, 2017). Another model that should be used more widely is the zebra fish, which can easily be modified by the morpholino technique (Newman et al., 2011, 2014; Caramillo and Echevarria, 2017; Drummond and Wisniewski, 2017). Analysis of human patient samples revealed several mutations and biomarkers that were indicative of the AD pathology (Bondi et al., 2017). Therefore, especially cerebrospinal fluid and blood are investigated to identify peripheral oxidatively modified proteins potentially involved in AD progression (Di Domenico et al., 2011). However, as summarized in (Drummond and Wisniewski, 2017), there is currently no model available replicating all features of human AD. As discussed above, Alzheimer's disease results from multiple pathogenic mechanisms and dysfunctions, one of which is the dysregulation of the redox homeostasis (von Bernhardi and Eugenín, 2012; Conrad et al., 2013). Brain organoids are a good approach since they are a model that presents a human proteomic and genomic background, with different cell types (neurons and astrocytes) organized in tissular structures. Nevertheless, they still lack some very important characteristics, such as all the cell types present in the human brain (Bi et al., 2021).

The *in vivo* analysis of redox modifications is very challenging due to the variety of possible and mostly transient modifications (Hanschmann, 2013). Proteome analysis using 2D-gel electrophoresis followed by mass spectrometry (MS) and/or computer-based analysis allows to compare samples and thereby to identify isoforms, mutants, stable protein interactions, and posttranslational modifications (Sultana et al., 2006a,b). To identify specifically oxidized proteins in human AD brain tissue the 2D-gel electrophoresis can be coupled to an immunochemical detection of protein carbonyl and nitrated proteins followed by mass spectrometry (Butterfield et al., 2006). However, the limitation of this method are membrane proteins, low abundant proteins, and proteins with high trypsin content, which cannot be solubilized or detected, respectively (Sultana et al., 2006b). An alternative are non-SDS-PAGE based methods coupled with MS analysis that are increasingly used. Lennicke et al. summarized gel-based and non-gel-based methods in general for the identification and enrichment of redox-modified proteins by redox proteomics in (Lennicke et al., 2016).

Monitoring the redox state *in vivo* requires specific biocompatible probes for different reactive species and, since their transient concentrations are usually very low, high sensitivity. Specific electron paramagnetic resonance (EPR) probes (aminoacyl radicals) are *in vivo* reporters to analyze the redox state of the brain and the blood-brain barrier integrity in a 5xFAD mouse model of AD (Vesković et al., 2021). A near-infrared emissive iridium probe is sensitive and selective toward peroxynitrite/glutathione redox cycles (Wu et al., 2021). Available small molecule near-infrared fluorescent probes are used to detect Tau, Aβ, and reactive species *in vivo*, e.g., CRANAD-61 and CRANAD-5 detect ROS at micro- and macro-levels in APP/PS1 mice (Fang et al., 2019). Numerous fluorescent probes for detection of varying redox active species (ROS/RNS/RSS) *in vivo/in cellulo* are summarized in (Lü, 2017) and (Wu et al., 2019). Luminescent lanthanide complexes selectively detect i.e., O_2_^•−^, ^•^OH, ^1^O_2_, and H_2_O_2_ in a μM to nM range (Galaup et al., 2021). Disadvantages of probes for the *in vivo* detection of redox reactive compounds may include artifactual amplification of the intensity by the formation of intermediate radicals, light sensitivity leading to artifactual H_2_O_2_ generation, reaction with other reactive species, and interference/dependency of the reaction on enzymatic activity (Kalyanaraman et al., 2012).

New tools to address the difficult analysis of redox modifications are being developed and optimized. Redox sensors measuring real time dynamic changes of e.g., H_2_O_2_ production in different compartments of the cell, are a powerful tool to analyze the impact of AD induced changes in the cellular redox state in 2D and 3D cell culture and animal models. However, the data analysis of redox modifications and measurements of ROS need to be very conscientious since commercially available kits bear challenges and limitations that too easily lead to misinterpretations (Murphy et al., 2022). In the future it will not only be important to identify the exact nature of reactive species of ROS/RNS/RSS but also the sources, as they could pose potential targets for new therapeutic approaches.

## Conclusion and future challenges

The emergence of novel experimental models, such as 3-dimensional cell cultures and complex transgenic animals, coupled with new imaging techniques and redox sensors promise great advances in the study of redox regulation in AD.

Many of the studies revised in the present work focus on the action of the Aβ peptide and the oligomers or plaques over the formation of different ROS and an increased oxidation of macromolecules. However, the central role of this peptide in AD has been questioned during the last two decades, changing the focus to other factors such as APP, Tau, its phosphorylation and the formation of neurofibrils. In the present bibliographical research, we were able to find scant works that put their whole attention on these proteins and their probable relation to redox regulation. It has been shown that APP presents different redox states and that these could be related to dimerization of the protein or its subproducts. Furthermore, 4R Tau possesses two Cys residues that are key for its aggregation. This protein has also been linked to mitochondrial changes, and this is of most importance, since many authors have found a clear relation between mitochondrial modifications, the production of O_2_^•−^ and H_2_O_2_, metabolic changes and the onset of AD. Thus, further investigations that focus not only on Aβ peptide, but on its precursor protein and Tau are of most interest in the field. Additionally, further investigations on alterations of metabolic pathways and redox metabolism are necessary to fully understand their interplay and importance in the development and progression of AD. Even small, post translational changes such as oxidative modifications regulate the function of biomolecules and can have a strong impact on the onset and progression of diseases linked to neurodegeneration and neuroinflammation. However, we found that many studies use unspecific markers and kits to analyze ROS and the oxidation of macromolecules. These tools can be quite misleading, and they give incomplete and inconsistent information. Future research will aim to understand redox regulation of specific proteins and pathways in neurodegenerative diseases, through specific second messengers, that need to be identified with certain detail. Thus, we believe that the use of novel, advanced and specific techniques promise a continuous progress in the untangling of the relations of AD and redox signaling and dys/regulation.

More research is needed to characterize the physiological function and the pathological impact of thiol switches in key proteins linked to AD, such as Aβ, Tau, and the interaction partners that regulate their function.

## Author contributions

All authors listed have made a substantial, direct, and intellectual contribution to the work and approved it for publication.

## Funding

We acknowledge the financial support to EMH from the Deutsche Forschungsgemeinschaft (Ha 8334/2-2) within the cooperative DFG program SPP1710: Dynamics of Thiol-based Redox Switches in Cellular Physiology). We acknowledge support for the Article Processing Charge by the German Research Foundation and the Open Access Publication Fund of the University of Greifswald.

## Conflict of interest

The authors declare that the research was conducted in the absence of any commercial or financial relationships that could be construed as a potential conflict of interest.

## Publisher's note

All claims expressed in this article are solely those of the authors and do not necessarily represent those of their affiliated organizations, or those of the publisher, the editors and the reviewers. Any product that may be evaluated in this article, or claim that may be made by its manufacturer, is not guaranteed or endorsed by the publisher.
